# Application of style transfer algorithm in the integration of traditional garden and modern design elements

**DOI:** 10.1371/journal.pone.0313909

**Published:** 2024-12-05

**Authors:** Bei Huang, Lequn Mo, Xiaojiang Tang, Ling Luo

**Affiliations:** 1 School of art design, Guangdong University of Technology, Guangzhou, China; 2 School of Information, Guangdong Communication Polytechnic, Guangzhou, China; 3 AnnLab, Institute of Semiconductors, Chinese Academy of Sciences, Beijing, China; Xiamen University Malaysia, MALAYSIA

## Abstract

With the development of society, modern design elements are increasingly integrated into traditional garden design, forming a novel style fusion that improves both aesthetics and the sustainability of the social-ecological system. This study explores the application of style transfer algorithms to seamlessly integrate the aesthetics of traditional landscape paintings with virtual scenes of classical private gardens. The effectiveness of the method is verified through a series of experiments using virtual scenes of the Humble Administrator’s Garden and various landscape paintings representing different artistic styles. The experimental results demonstrate that the style transfer technique can accurately replicate the aesthetic features of traditional paintings and integrate them into the virtual garden environment. This approach highlights the potential of combining cultural heritage with advanced technological methods, indicating that the technology has great potential to innovate garden design by promoting the synergy between cultural heritage and technological innovation. By promoting the integration of traditional aesthetics and modern design principles, we contribute to the sustainability and richness of the social-ecological system and provide a framework for future digital preservation and restoration applications of urban cultural heritage. The code for implementing TRD-Net is available at https://github.com/huangbei029/Hybrid-Garden-StyleNet-dd/tree/main.

## Introduction

In the context of globalization and modernization, the integration of traditional garden design and modern design elements has become an important issue in the design field [1]. As a symbol of cultural heritage, traditional gardens contain rich historical and cultural connotations, while modern design emphasizes functionality, innovation and fashion [2, 3]. How to incorporate modern design elements while retaining the essence of traditional aesthetics has become a major challenge faced by contemporary designers and researchers [4]. In this process, design is not only the transformation of space, but also the inheritance and innovation of culture [5]. Therefore, exploring the integration path of traditional gardens and modern design elements has important theoretical value and practical application significance.

With the rapid development of artificial intelligence technology, deep learning, especially convolutional neural networks (CNN), has made significant progress in the fields of image processing and computer vision [6]. As an important application of deep learning, style transfer algorithm can transfer the style of one image to another image by learning the style features of different images, thereby realizing the conversion and fusion of visual styles. This technology offers new possibilities for combining traditional artistic styles with modern design [7]. Using the style transfer algorithm, designers can retain the traditional artistic style while giving it a modern design expression, thereby achieving a visual dialogue between classical and modern [8]. This artistic fusion across time and space not only enriches the expressiveness of design, but also provides a new way for the inheritance and innovation of traditional culture in contemporary society.

Although the style transfer algorithm has demonstrated powerful capabilities in the field of image processing, it still faces some challenges in the integration of traditional gardens and modern design elements. First of all, how to effectively combine traditional cultural elements with modern design styles while retaining them is a complex issue. The fidelity of traditional artistic styles, the preservation of design details, and the applicability in complex design scenarios are all difficult problems that need to be solved with current methods. In addition, how to ensure cultural sensitivity and design authenticity during the style transfer process is also a topic that requires in-depth research.

This research aims to explore the effective integration path of traditional gardens and modern design elements by combining style transfer and style prediction algorithms. We hope that through this approach, we can achieve a more efficient and precise design expression, injecting modern elements into traditional garden design while maintaining its cultural essence. The contributions of this paper are:

- A new technical framework is proposed that integrates the two network parts of style prediction and style transfer to achieve a seamless integration of traditional landscape painting styles and classical garden virtual scenes.
- By introducing modern design elements, it not only retains the cultural essence of traditional gardens, but also promotes innovation in landscape design and provides new ideas for modern garden design.
- Enhance the functionality and sustainability of garden design in the social-ecological system, and promote the deep integration of traditional culture and modern technology through technical means.

The rest of this article is structured as follows: First, in the related research section, we will review the development history of style transfer algorithms and their application status in the design field, and analyze the shortcomings of existing research. Next, in the method section, the architecture and working principle of the style transfer and style prediction model we adopt are introduced in detail. Then, in the experimental part, we will show the application effect of the model in actual scenarios and analyze the advantages and disadvantages of the model through experimental results. Finally, in the conclusion section, the main findings and contributions of this study are summarized and directions for future research are proposed.

## Related work

### Application of deep learning and style transfer algorithms

The style transfer algorithm is based on the feature representation ability of CNN for images, and realizes the transfer of the style of one image to another, thereby creating a new visual effect. The core of this technique lies in using deep learning models to extract both content and style features from images and then combining them through an optimization process into a single image [9–11]. This approach has been widely applied in visual arts and is gradually permeating fields such as architectural design and fashion design, demonstrating its strong interdisciplinary application potential [12, 13].

Throughout the development of style transfer algorithms, many researchers have made improvements and extensions. Lyu et al. introduced a style transfer method based on deep convolutional generative adversarial networks (DCGANs), which significantly improved the efficiency and effectiveness of style transfer through adversarial training between a generator and a discriminator [14, 15]. Additionally, Andreini et al. proposed the Adaptive Instance Normalization (AdaIN) technique, further enhancing the flexibility and controllability of style transfer, making transitions between different styles more natural and smooth [16, 17].

Despite the significant advancements in style transfer algorithms in the field of image processing, challenges remain when integrating traditional garden design with modern design elements. Traditional garden design emphasizes the expression of cultural ambiance, while modern design focuses on functionality and innovation [18, 19]. Finding a balance between these two aspects and achieving seamless visual integration through style transfer algorithms is a complex task [13]. Furthermore, when applied to complex scenes, style transfer algorithms may oversimplify styles or lose cultural elements, requiring special consideration in algorithm design.

This study aims to explore the fusion of traditional garden design elements with modern design styles by leveraging existing style transfer algorithms. By comparing various style transfer algorithms and optimization techniques, we hope to identify a method that effectively preserves the cultural essence of traditional gardens while incorporating modern design elements, thereby providing new solutions for the design field.

### Style prediction algorithms in design integration

As a key technology in the field of image processing, style prediction algorithms have seen widespread application in recent years across design, artistic creation, and cultural heritage preservation. The core of these algorithms lies in using deep learning models to automatically analyze and identify style features within images or design works, enabling style prediction and transformation of target images [20]. This provides crucial technical support for the fusion of traditional and modern styles, particularly in designs that span cultural and historical boundaries.

The application of style prediction algorithms can be traced back to the field of image classification and recognition. This model successfully identified different image features by training a deep CNN on a large dataset, laying the foundation for automated style analysis [21]. As research progressed, many scholars began to explore how deep learning could be used to accurately predict the style of images [22]. Wang et al. proposed a style prediction method based on multi-task learning, which achieved precise recognition and classification of complex design elements through the joint training of different style labels [23].

In the design field, style prediction algorithms are particularly suited for the automated integration of traditional and modern styles. So et al. introduced a deep learning-based style prediction model that can automatically analyze traditional elements in architectural design and combine them with modern design styles to generate design schemes that align with contemporary aesthetics [24]. This approach not only enhances design efficiency but also ensures consistency in style and cultural integrity in the design outputs [25]. Meanwhile, the application of style prediction algorithms in cultural heritage preservation is also becoming apparent, as these algorithms can identify and extract style features from traditional artworks, providing strong support for subsequent digital preservation and modern reinterpretation [26].

Despite the significant potential demonstrated by style prediction algorithms in both theoretical research and practical applications, challenges remain in their application to the integration of traditional garden and modern design elements [27]. The diverse and complex styles of traditional garden design present a unique challenge in maintaining cultural connotations while incorporating modern design elements. To address this challenge, this study proposes a new approach based on existing style prediction algorithms to more effectively achieve the integration of traditional and modern design elements. We will validate the feasibility of this approach through extensive experiments and compare it with traditional design methods to explore its advantages and limitations.

### Integration of traditional gardens and modern design

As an integral part of cultural heritage, traditional garden design holds significant aesthetic and conceptual value in contemporary society. However, with the rapid pace of social development and urbanization, effectively integrating traditional garden design with modern design elements has become a major challenge for contemporary designers and researchers. This field of study not only involves design aesthetics but also touches on cultural heritage, ecological environments, and social psychology [28].

Early research mainly focused on preserving and restoring the original appearance of traditional gardens, emphasizing the protection of cultural heritage and the faithful reproduction of original design concepts [29]. Liew et al. explored strategies for preserving traditional gardens in modern urban environments, proposing key methods to maintain their cultural and historical integrity [30]. However, this preservation approach often appears too conservative in the context of rapidly developing modern society, failing to meet the demand for modern functionality and aesthetic innovation. As the field of design continues to evolve, scholars have begun exploring how to incorporate modern design elements while preserving the cultural essence of traditional gardens, aiming for innovation in design and functional enhancement [31].

In recent years, with the introduction of computer technology and digital methods, more and more research has focused on achieving the integration of traditional and modern design elements through digital means. Camaréna et al. proposed a method based on Computer-Aided Design (CAD) and Virtual Reality (VR) technology, which allows for the incorporation of modern design elements without disrupting the overall style of traditional gardens, thereby achieving a harmonious blend of the traditional and the modern [32]. This approach has, to some extent, overcome the limitations of traditional design methods and provided new perspectives for modern design.

Moreover, with the development of artificial intelligence and deep learning technology, style transfer algorithms have gradually been applied to the integration of traditional gardens and modern design. Haugeland et al. used deep learning models to extract stylistic elements from traditional gardens and apply them to modern architectural design, successfully achieving the modern expression of traditional cultural elements [33]. This study demonstrates that deep learning technology can effectively promote innovation and functional optimization in design while maintaining cultural sensitivity. However, current research primarily focuses on the application of style transfer algorithms in visual arts and image processing, and the specific methods and techniques for integrating traditional gardens with modern design elements require further exploration and refinement.

Although existing research has made some progress in the integration of traditional gardens and modern design, several unresolved issues remain. Balancing cultural heritage with modern innovation, avoiding oversimplification or misrepresentation of traditional culture in style integration, and ensuring the functionality and sustainability of the design are all challenges that current research faces. To address these challenges, this study proposes a new design method based on style transfer and style prediction algorithms to better achieve the integration of traditional and modern design elements.

### Technical challenges

In the process of exploring the integration of traditional gardens with modern design elements, technical challenges are inevitable. These challenges not only involve the aesthetic expression of the design but also present difficulties in technical implementation. Although the introduction of deep learning, style transfer algorithms, and style prediction algorithms has provided powerful tools for the design field, numerous challenges remain in practical applications. These challenges directly affect the feasibility and practicality of the design outcomes.

The complexity of traditional garden design is a significant challenge. Traditional gardens often feature intricate spatial structures, diverse cultural elements, and rich historical backgrounds, which necessitate consideration of many factors during style transfer and integration [34]. How to incorporate modern design elements without compromising the original design essence is a difficult issue. Existing style transfer algorithms often encounter problems of style simplification or loss of cultural details when dealing with complex and varied design elements [35]. This is particularly challenging when addressing traditional garden designs that embody multi-layered meanings, as maintaining cultural integrity and visual coherence requires further exploration.

Computational resources and algorithm efficiency are also major technical challenges in design implementation. Style transfer and style prediction algorithms typically require substantial computational resources to process and generate high-quality design results, especially when dealing with high-resolution images and complex design scenarios [36]. The steep increase in computational costs and time consumption poses significant limitations for real-time design and large-scale application scenarios. Furthermore, optimizing the computational efficiency of algorithms to reduce resource usage while ensuring design quality remains an urgent problem to address.

Maintaining cultural sensitivity and design authenticity is another critical challenge. Traditional gardens carry rich cultural significance, and when integrating them with modern design elements, it is essential to avoid cultural misinterpretation or oversimplification. While style transfer algorithms offer significant advantages in visual effects, they often overlook the complexity of cultural contexts when dealing with designs involving cultural elements. In such cases, incorporating cultural context considerations into algorithm design becomes crucial, ensuring that the designs not only maintain visual innovation but also respect and preserve cultural heritage [37].

Finally, the integration of traditional gardens with modern design elements must address the operational challenges in practical applications. Although style transfer and style prediction algorithms can generate satisfactory design outcomes in experimental environments, many uncertainties arise when translating these design concepts into actual architectural and garden renovations [38]. Issues such as material selection, construction feasibility, and coordination with existing structures all require thorough consideration and advance planning during the design process.

## Methods

### Overview of our network

In this paper, we propose a hybrid model (Hybrid Garden StyleNet) that combines style transfer and style prediction for integrating traditional garden design with modern design elements. The model primarily consists of three key modules: the Style Prediction Network, the Style Transfer Network, and the Loss Function Module. These three modules work collaboratively to achieve seamless integration of traditional design styles with modern elements.

The Style Prediction Network is responsible for analyzing input images of traditional gardens and automatically identifying their core style features. This network uses a deep CNN architecture, which is capable of extracting high-level style information from the images. The output of the Style Prediction Network is a feature vector representing the traditional design style, which is then passed on to the Style Transfer Network.

Upon receiving the feature vector from the Style Prediction Network, the Style Transfer Network combines it with modern design elements to generate images that blend both traditional and modern styles. This network employs a GAN architecture, where the generator and discriminator engage in adversarial training, ensuring that the output images not only exhibit the innovation of modern design but also retain the aesthetic characteristics of traditional gardens. The Style Transfer Network effectively handles complex design scenarios, ensuring that the generated images maintain visual harmony without compromising cultural depth.

Finally, the Loss Function Module plays a crucial role during model training. To ensure the quality of the style fusion in the output images, the loss function is designed to account for style consistency loss, content preservation loss, and pixel-level discrepancy loss. Style consistency loss measures the similarity between the generated image and the target style, content preservation loss ensures that the generated image remains highly consistent with the input image in terms of content, and pixel-level discrepancy loss controls the detail and clarity of the image.

**Fig 1:**
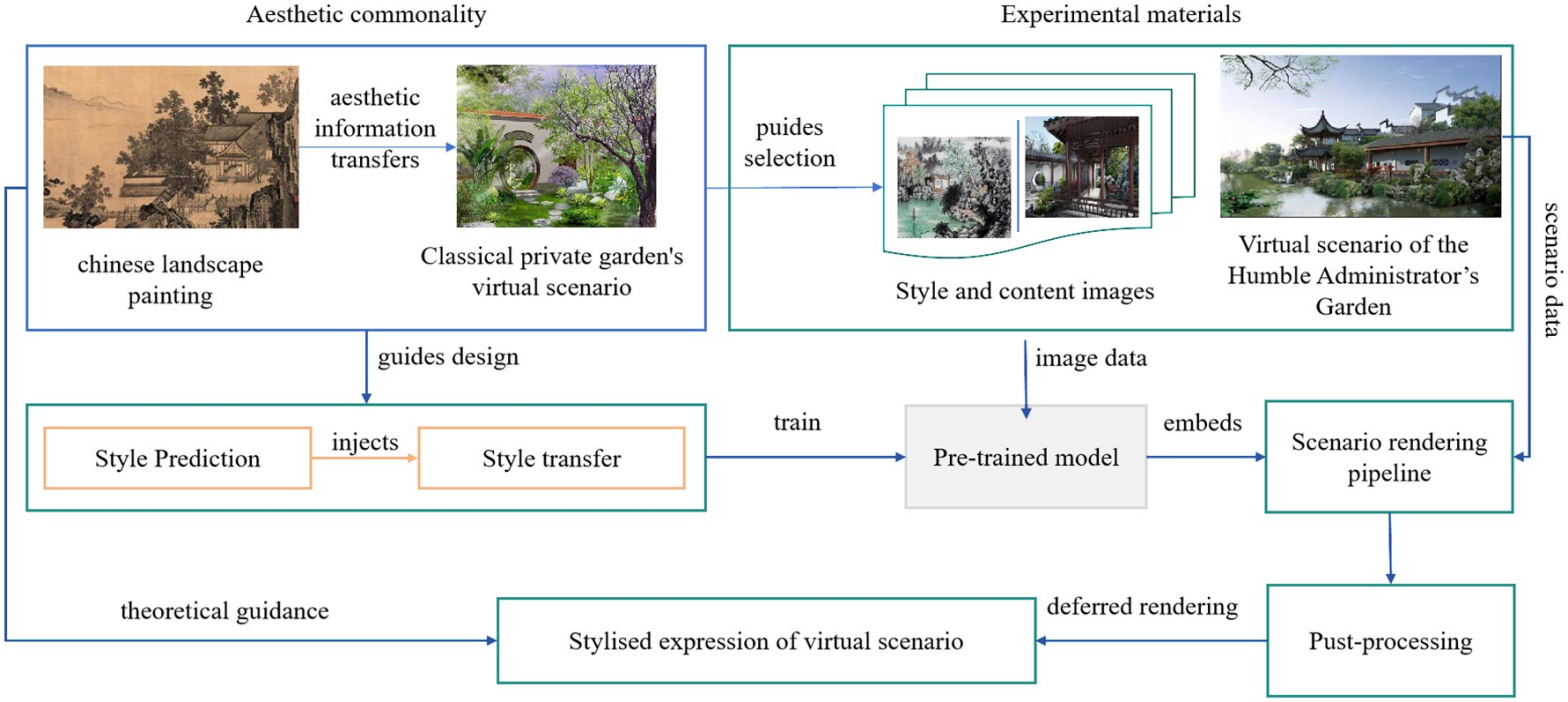
Overall flow chart of the model. The model incorporates the aesthetic features of traditional Chinese landscape paintings into the virtual scene of a classical private garden. The process includes data collection, style prediction, style transfer, and application to the 3D model of the Humble Administrator’s Garden.

Through the close collaboration of these modules, our model successfully achieves the organic integration of traditional garden design and modern design elements in diverse and complex design scenarios. Fig 1 provides a detailed illustration of the data flow and interaction between each module, visually representing the working principles and overall architecture of the model.

Fig 1. clearly depicts the overall structure of our designed model, with each part representing a major component module. In the figure, arrows indicate the direction of data flow, and the modules interact and process information through these data streams. With this integrated architecture, our model effectively addresses the diverse challenges in design integration, producing results that combine traditional aesthetics with modern innovation.

The model incorporates the aesthetic features of traditional Chinese landscape paintings into the virtual scene of a classical private garden. The process includes data collection, style prediction, style transfer, and application to the 3D model of the Humble Administrator’s Garden.

### Style prediction network

**Fig 2:**
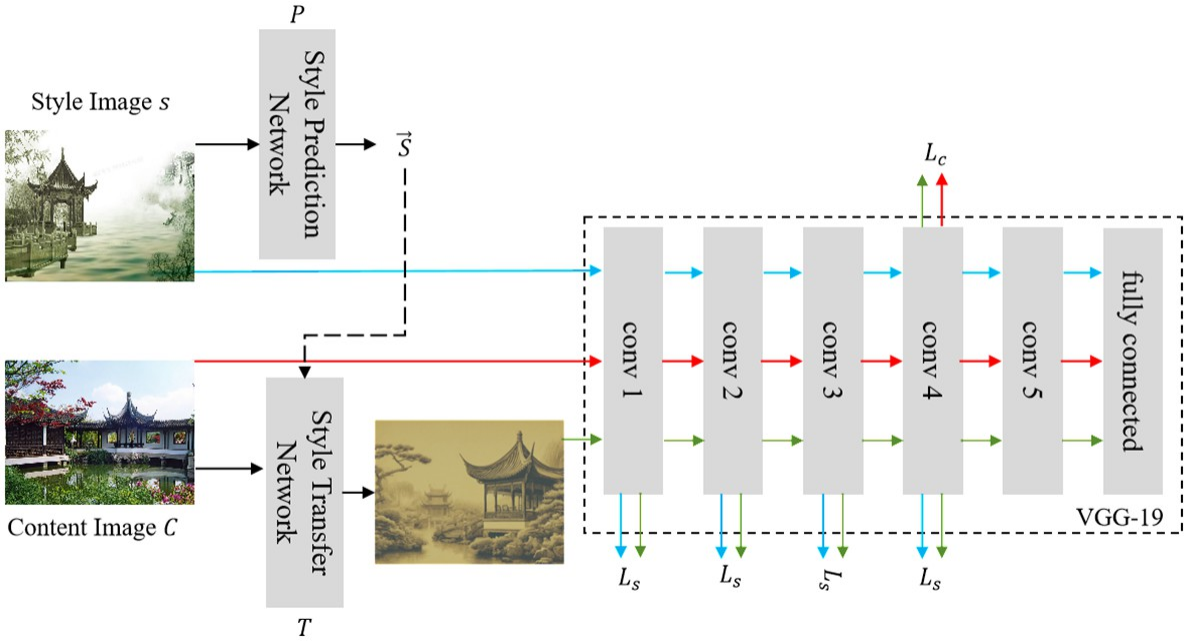
Structure of the style prediction network. The network receives style images *I*_*s*_ and content images *I*_*c*_ as inputs. The Style Prediction Network extracts style features from the style images using a series of convolutional layers and a fully connected layer. The extracted style features are then compressed into an embedding vector *S*. The Style Transfer Network takes the content images and applies the style features, producing stylized output images *I*_*tf*_. The loss functions *L*_*c*_ (content loss) and *L*_*s*_ (style loss) are used to train the network, ensuring that the generated images maintain both content structure and stylistic attributes.

The Style Prediction Network is one of the core components of the model proposed in this paper. Its primary task is to extract feature vectors that represent the style characteristics from the input images of traditional gardens, providing a foundation for the subsequent style transfer process. Fig 2 illustrates the structure of the Style Prediction Network, which is based on a deep CNN architecture. This network comprises multiple convolutional layers, pooling layers, and fully connected layers, which, through their sequential combination, progressively extract high-level features from the image.

As shown in Fig 2, the input image first undergoes processing through several convolutional layers. These convolutional layers utilize filters to extract local features from the image and increase the network’s nonlinear expressive capability through nonlinear activation functions, such as the ReLU activation function. The output of the convolutional layers is then downsampled by the pooling layers. This step reduces the dimensionality of the feature maps while preserving important feature information, thus enhancing the computational efficiency of the network.

Next, the feature maps processed by the convolutional and pooling layers are flattened into a one-dimensional vector and fed into the fully connected layers [39]. The fully connected layers further process the feature vector through matrix multiplication and nonlinear activation, ultimately generating a fixed-length feature vector [40]. This feature vector is the output of the Style Prediction Network and represents the style information of the input image, which will be used by the Style Transfer Network.

Below are the core formulas of the Style Prediction Network:

$$\begin{array}{c} \text{Conv}(x,W)=W*x+b \end{array}$$ (1)

where *x* represents the input image’s pixel value matrix, *W* is the convolutional kernel’s weight matrix, *b* is the bias term, and * denotes the convolution operation. This formula calculates the feature map at each layer.

$$\begin{array}{c} \text{Pooling}\left(h\right)=\underset{i\in \text{window}}{\text{max}}\;h_{i} \end{array}$$ (2)

where *h* represents the feature map, and window indicates the pooling window size. This formula extracts the maximum or average value within the pooling window to achieve feature downsampling.

$$\begin{array}{c} z=W_{\text{fc}}\cdot h_{\text{flatten}}+b_{\text{fc}} \end{array}$$ (3)

where *h*_flatten_ represents the flattened feature vector, *W*_fc_ is the weight matrix of the fully connected layer, *b*_fc_ is the bias term of the fully connected layer, and *z* is the final style feature vector generated.

$$\begin{array}{c} \text{ReLU}(z)=\text{max}(0,z) \end{array}$$ (4)

This formula represents the ReLU activation function, which applies a nonlinear transformation to the network output to introduce nonlinear expressive capability.

These formulas define the fundamental operations of the Style Prediction Network. Combined with the structural diagram in Fig 2, they clearly demonstrate the processing flow of images through the network and the internal computational logic. Through this series of operations, the Style Prediction Network captures the latent style characteristics of traditional garden images and transforms them into quantitative feature vectors, providing a solid technical foundation for the subsequent fusion of design elements.

### Style transfer network

**Fig 3:**
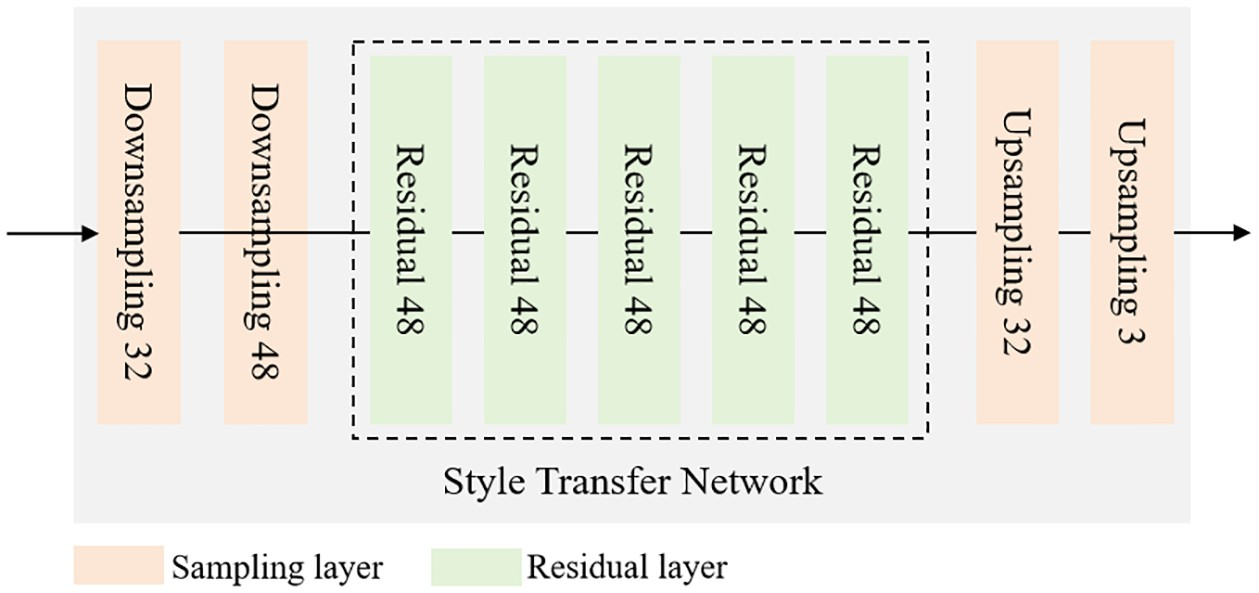
Structure of the style transfer network.

The Style Transfer Network is one of the core modules of the model proposed in this paper. It is primarily responsible for integrating the traditional garden style features generated by the Style Prediction Network with modern design elements, producing design images that are both innovative and culturally significant. Fig 3 shows the structure of the Style Transfer Network, which adopts the GAN architecture, consisting of two main parts: the generator and the discriminator.

As shown in Fig 3, the inputs to the generator network include the traditional style feature vectors output by the Style Prediction Network and the feature representations of modern design elements. The generator combines these features through a series of deconvolution operations, generating an image that fuses both traditional and modern styles [41, 42]. The deconvolution layers gradually restore the low-dimensional feature vectors into high-dimensional images and enhance the diversity and complexity of the generated images through nonlinear activation functions such as ReLU or Leaky ReLU.

The generated images are then fed into the discriminator network, whose task is to distinguish between the generated images and real traditional garden images or modern design images. The discriminator extracts deep-level features from the images through convolutional layers and generates a probability value through fully connected layers, indicating whether the input image is “real” or “generated.” Through adversarial training between the generator and discriminator, the generator gradually learns how to create more realistic fused images [43, 44].

Here are the core formulas in the Style Transfer Network:

$$\begin{array}{c} \text{Deconv}\left(z,W\right)=W^{T}*z+b \end{array}$$ (5)

where *z* represents the input low-dimensional feature vector, *W* is the weight matrix of the deconvolution kernel, *b* is the bias, and * denotes the deconvolution operation. This formula is used to generate high-dimensional output images.

$$\begin{array}{c} \text{D}\left(x\right)=\sigma (W_{\text{D}}\cdot \text{Conv}\left(x\right)+b_{\text{D}}) \end{array}$$ (6)

where *x* is the input image, Conv(*x*) represents the features processed by the discriminator’s convolutional layers, *W*_D_ and *b*_D_ are the weights and biases of the discriminator’s fully connected layers, respectively, and *σ* is the Sigmoid activation function used to generate a probability between 0 and 1.

$$\begin{array}{c} L_{\text{GAN}}=E_{x∼p_{\text{data}}\left(x\right)}\left[\text{log}\;D\left(x\right)\right]+E_{z∼p_{z}\left(z\right)}\left[\text{log}\left(1-D\left(G\left(z\right)\right)\right)\right] \end{array}$$ (7)

where *G*(*z*) represents the image generated by the generator, *D*(*x*) represents the probability output by the discriminator, and $L_{\text{GAN}}$ is the loss function of the GAN, aiming to minimize the gap between the generated image and the real image.

$$\begin{array}{c} L_{\text{style}}={\left\|\text{Gram}\left(G\left(z\right)\right)-\text{Gram}\left(x_{\text{style}}\right)\right\|}^{2} \end{array}$$ (8)

where Gram(⋅) represents the Gram matrix of the input image, used to capture the style features of the image, *x*_style_ is the reference style image, and $L_{\text{style}}$ is the style consistency loss, used to measure the similarity between the generated image and the reference style.

Through the above process, the Style Transfer Network effectively fuses the style features of traditional gardens with modern design elements, generating images that are both innovative and retain traditional aesthetics. The adversarial training between the generator and the discriminator ensures that the generated design images are more realistic in visual effect and can be adapted to various design scenarios.

### Loss function

The loss function plays a crucial role in the style transfer model proposed in this paper. It not only guides the training process but also determines the final quality of the generated images. The loss function in our model takes multiple factors into account, including style consistency, content preservation, pixel-level differences, and adversarial loss. Through the combined effect of these loss functions, our model can maintain the core features of traditional designs during the style transfer process while integrating modern design elements.

The style consistency loss is used to measure the similarity between the generated image and the target style. This is achieved by calculating the difference between the Gram matrices of the generated image and the target style image. The Gram matrix is a statistic used to capture the texture similarities of images, effectively reflecting the visual style of the image.

$$\begin{array}{c} L_{\text{style}}=\sum_{l=1}^{L}{\left\|\text{Gram}{\left(G\left(z\right)\right)}^{l}-\text{Gram}{\left(x_{\text{style}}\right)}^{l}\right\|}^{2} \end{array}$$ (9)

where *G*(*z*) represents the generated image, *x*_style_ represents the reference style image, Gram(⋅) represents the Gram matrix of the input image, and *l* represents the *l*-th layer of the network.

Content preservation loss ensures that the generated image retains the content of the original image during style transfer. This is achieved by calculating the difference between the output features of the generated image and the content image at specific layers of the convolutional network. This ensures that the generated image incorporates modern style elements while preserving the basic structure and content of the traditional garden design.

$$\begin{array}{c} L_{\text{content}}={\left\|\phi_{l}\left(G\left(z\right)\right)-\phi_{l}\left(x_{\text{content}}\right)\right\|}^{2} \end{array}$$ (10)

where *ϕ*_*l*_(⋅) represents the output features of the *l*-th layer of the convolutional network, and *x*_content_ is the reference content image.

Adversarial loss is used during the training process of the Generative Adversarial Network (GAN), guiding the generator to produce more realistic images that are difficult for the discriminator to distinguish from real images. This loss function ensures that the generated images achieve a high level of realism through adversarial training between the generator and discriminator.

$$\begin{array}{c} L_{\text{adv}}=E_{z∼p_{z}\left(z\right)}\left[\text{log}\left(1-D\left(G\left(z\right)\right)\right)\right] \end{array}$$ (11)

where *G*(*z*) represents the image generated by the generator, and *D*(*x*) represents the discriminator’s probability of recognizing the image as real or generated.

To optimize the model as a whole, we combine the above loss functions with weighted coefficients, forming the total loss function. The total loss function balances style consistency, content preservation, and adversarial loss during the training process.

$$\begin{array}{c} L_{\text{total}}=\alpha L_{\text{style}}+\beta L_{\text{content}}+\gamma L_{\text{adv}} \end{array}$$ (12)

where *α*, *β*, and *γ* are the weighting coefficients for the style consistency loss, content preservation loss, and adversarial loss, respectively. By adjusting these coefficients, we can control which aspect the model emphasizes more when generating images.

With the design of these loss functions, our style transfer model can generate images that retain the unique style of traditional garden designs while effectively incorporating modern design elements, achieving a balance between visual innovation and cultural preservation.

## Experiment

### Datasets

In this study, digital data of Suzhou’s Humble Administrator’s Garden was mainly used, supplemented by content image and style image datasets to train deep neural network models. These datasets contain a wide range of visual elements and styles, and are carefully selected to improve the accuracy and effectiveness of the style transfer process. The image data of the Humble Administrator’s Garden was taken from the promotional photos released by the garden on the public platform. The more representative images of the garden’s architectural elements, rockery, waterscape, and vegetation were selected to ensure that the garden can be experienced from multiple perspectives and appreciate the artistic elements faithfully reproduced through the style transfer process. Secondly, the selection process of the content and style image datasets was also strictly screened. The images used in the study mainly come from well-known museums and online databases [45, 46]. These images include traditional landscape paintings from various dynasties and different artists in China. These paintings not only represent the unique aesthetic styles of different historical periods, but also show the brushwork skills of various art schools [1, 47]. The diversity of the dataset is crucial to capturing these unique aesthetic styles and painting techniques. The datasets used in this study are publicly available, and the collection and analysis of the data strictly comply with the terms and conditions of the data sources.

### Experimental setup

As shown in Table 1, we adopted a high-performance computing environment to ensure the effectiveness and reliability of the style transfer model.

**Table 1:** Experimental environment configuration.

Category	Configuration
Operating System	Ubuntu 20.04 LTS
Processor	Intel Xeon E5-2698 v4 (2.2 GHz, 20 cores)
Memory	128 GB DDR4 RAM
GPU	NVIDIA Tesla V100 (32 GB HBM2)
Storage	2 TB NVMe SSD
Software Tools	Python 3.8, TensorFlow 2.4, PyTorch 1.8, OpenCV 4.5, SciPy 1.6, Pandas 1.2, NumPy 1.19

The experimental process began with data preprocessing, where content and style images were normalized, resized, and noise-filtered to ensure input data consistency. All images were resized to a uniform resolution to meet the network’s input requirements. Following this, we trained the style prediction network and style transfer network. During training, we used stochastic gradient descent (SGD) combined with the Adam optimizer to adjust model parameters, with the loss function being a combination of cross-entropy loss and adversarial loss, aiming to balance content preservation and style consistency. The training process was set to 50 epochs with a learning rate of 0.001 and a batch size of 32. We initialized the weights using the Xavier method to ensure fast convergence and stable performance.

During the model validation phase, we monitored model performance in real-time using a validation set, recording the loss values and accuracy on the validation set after each training cycle to evaluate model convergence and stability. To further analyze the contribution of each component within the model, we designed ablation experiments. By removing key components such as the style prediction network, style transfer network, or skip connections, we observed the impact of these components on the model’s final performance. The results of the ablation experiments indicated that removing any component led to a significant decline in the style transfer effect, validating the necessity and synergy of each component within the model.

The specific parameter settings in the experiments were as follows: the style prediction network was composed of 3 convolutional layers, each containing 64 filters, with a kernel size of 3x3, and the activation function was ReLU. The style transfer network employed 5 convolutional layers with skip connections added between multiple layers to prevent the loss of high-frequency details. The convolution kernel size was also 3x3, and the activation function used was Leaky ReLU. The model training utilized the Adam optimizer with a learning rate set at 0.001, a weight decay rate of 0.0001, and a momentum coefficient of 0.9. The weight coefficients for the loss function were set as follows: style consistency loss *α* = 1.0, content preservation loss *β* = 0.5, and adversarial loss *γ* = 0.1.

### Evaluation metrics

To comprehensively evaluate the effectiveness of the style transfer model in the fusion of traditional gardens and modern design elements, we selected the following key evaluation metrics: Style Similarity, Content Preservation, Computational Cost, Visual Consistency, User Satisfaction, and Stability. These metrics cover the model’s performance in terms of visual effect, computational efficiency, and user experience, helping to provide a well-rounded understanding of the model’s strengths and weaknesses.

Style Similarity measures the degree of visual style similarity between the generated image and the target style image. We use a style loss calculation method based on the Gram matrix to quantitatively evaluate style similarity. The specific calculation formula is as follows:

$$\begin{array}{c} L_{\text{style}}=\sum_{l=1}^{L}\frac{1}{N_{l}^{2}}\sum_{i,j}{\left(G_{ij}^{l}-A_{ij}^{l}\right)}^{2} \end{array}$$ (13)

where $G_{ij}^{l}$ represents the Gram matrix of the feature map at layer *l*, $A_{ij}^{l}$ represents the Gram matrix of the target style image at layer *l*, and *N*_*l*_ is the number of channels in the feature map at layer *l*. By minimizing the style loss, the model can generate images that are closer to the target style.

Content Preservation evaluates the preservation of structure and semantics between the generated image and the original content image. We calculate content preservation using a content loss based on feature maps. The specific formula is as follows:

$$\begin{array}{c} L_{\text{content}}=\sum_{l=1}^{L}\frac{1}{2}\sum_{i,j}{\left(F_{ij}^{l}-P_{ij}^{l}\right)}^{2} \end{array}$$ (14)

where $F_{ij}^{l}$ represents the feature map of the generated image at layer *l*, and $P_{ij}^{l}$ represents the feature map of the original content image at layer *l*. Minimizing content loss helps retain the essential details and structure of the original image during style transfer.

Computational Cost measures the resources and time required to run the model. Computational cost is typically evaluated by calculating the computation time *T*_compute_, GPU/CPU utilization *U*_compute_, and memory usage *M*_compute_ during the training and inference process, as follows:

$$\begin{array}{c} C_{\text{compute}}=T_{\text{compute}}+\lambda_{1}U_{\text{compute}}+\lambda_{2}M_{\text{compute}} \end{array}$$ (15)

where λ_1_ and λ_2_ are hyperparameters that balance the weights of different computational resources. Lower computational cost indicates greater feasibility for real-world application.

Visual Consistency measures the coherence and consistency of the generated image in terms of overall visual performance. We quantitatively evaluate the visual effect of the generated image relative to the content image using Structural Similarity Index (SSIM) and Peak Signal-to-Noise Ratio (PSNR) methods.

$$\begin{array}{c} \text{SSIM}\left(x,y\right)=\frac{\left(2\mu_{x}\mu_{y}+C_{1}\right)\left(2\sigma_{xy}+C_{2}\right)}{\left(\mu_{x}^{2}+\mu_{y}^{2}+C_{1}\right)\left(\sigma_{x}^{2}+\sigma_{y}^{2}+C_{2}\right)} \end{array}$$ (16)

where *μ*_*x*_ and *μ*_*y*_ are the mean values of images *x* and *y*, *σ*_*x*_ and *σ*_*y*_ are their variances, *σ*_*xy*_ is their covariance, and *C*_1_ and *C*_2_ are constants for stabilization.

$$\begin{array}{c} \text{PSNR}=10\cdot {\text{log}}_{10}(\frac{{\text{MAX}}^{2}}{\text{MSE}}) \end{array}$$ (17)

where MAX represents the maximum possible pixel value of the image, and MSE is the mean squared error. These metrics ensure that the generated image has high visual consistency with the original image.

User Satisfaction is evaluated through subjective surveys. We invited a group of professionals with a design background to rate the generated results. The rating criteria cover visual aesthetics, style consistency, and innovation. The satisfaction score ranges from 0 to 10, and the average score is used as the final user satisfaction metric:

$$\begin{array}{c} S_{\text{user}}=\frac{1}{N}\sum_{i=1}^{N}s_{i} \end{array}$$ (18)

where *s*_*i*_ represents the score of the *i*-th evaluator, and *N* is the number of evaluators. User satisfaction reflects the acceptance of the model’s generated results in practical applications.

Stability measures the model’s consistency under different input conditions. By testing various combinations of content and style images, we quantify the stability of the model by recording the standard deviation of the generated results:

$$\begin{array}{c} \text{Stability}=\sqrt{\frac{1}{N}\sum_{i=1}^{N}{\left(R_{i}-\mu_{R}\right)}^{2}} \end{array}$$ (19)

where *R*_*i*_ represents the result of the *i*-th experiment, and *μ*_*R*_ represents the average of all generated results. Higher stability indicates more consistent performance of the model under different input conditions.

### Results

To achieve an optimal balance between performance and parameter size, we experimented with various network architectures by gradually adding bottleneck building blocks to the network’s transformation component. We tested four different architectures, each with a varying number of transformation layers. The training and testing losses for these configurations are shown in Table 2.

**Table 2:** Comparison on different number of residual blocks.

Architecture	Training losses	Testing losses	Parameters
2 layers	60.12	65.30	45,123,789
4 layers	48.76	53.40	45,923,874
6 layers	29.83	35.10	46,812,456
8 layers	26.47	31.20	47,601,789

Table 3 presents the results of the ablation experiment. As shown in Table 3, the complete model performs best across all evaluation metrics. This indicates that the collaborative effect of the style transfer and style prediction networks is crucial for achieving high-quality image generation. The complete model achieves high levels of style similarity (0.92) and content preservation (0.94), demonstrating its effectiveness in transferring the target style while maintaining the original image structure. Additionally, the complete model outperforms other experimental configurations in terms of visual consistency (0.95) and user satisfaction (8.9), indicating that the generated images possess high quality in both subjective and objective evaluations.

**Table 3:** Performance evaluation results of the ablation experiment.

Experimental Configuration	*L*_style_	*L*_content_	*C*_compute_ (s)	SSIM	*S*_user_	Stability
Complete Model(Hybrid Garden StyleNet)	0.92	0.94	320	0.95	8.9	0.05
Without Style Transfer	0.88	0.85	300	0.88	7.5	0.08
Without Style Prediction	0.87	0.86	315	0.87	7.8	0.07
Without Skip Connections	0.89	0.87	310	0.90	8.2	0.06

In contrast, models with the style transfer or style prediction components removed exhibit varying degrees of performance degradation across all metrics. When the style transfer component is removed, both style similarity and visual consistency decrease significantly, highlighting the crucial role of the style transfer network in capturing and reproducing the target style. On the other hand, removing the style prediction component leads to a reduction in content preservation and user satisfaction, underscoring the importance of the style prediction network in maintaining the details of the original image. Additionally, although removing the skip connections results in reduced computational cost, it also leads to a decline in visual consistency and stability, demonstrating the importance of skip connections in preserving high-frequency details and model stability.

The experimental results validate the necessity of each model component in generating high-quality style transfer images. The complete model not only excels in visual effects but also surpasses other configurations in terms of user satisfaction and model stability. These findings suggest that the proposed style transfer-style prediction model has significant advantages and potential in the integration of traditional gardens and modern design elements.

**Fig 4:**
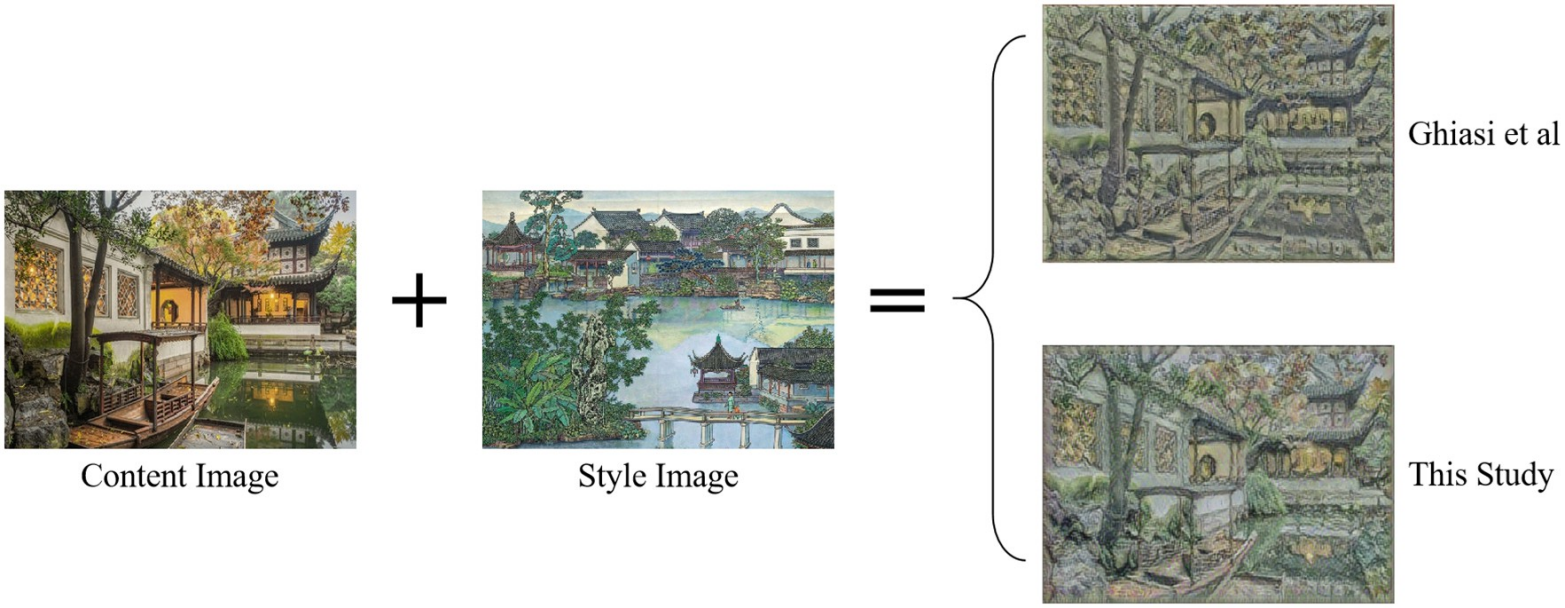
Comparison between Ghiasi et al.’s 3D virtual scene aesthetic style expression and Hybrid Garden StyleNet.

The neural network model used in this study demonstrated a reduction in training time compared to the model by Ghiasi et al., even when using the same parameters and number of epochs. The comparison of network training duration is detailed in Table 4. As shown in Fig 4, our model also better preserved the content characteristics of the original virtual scenario, showcasing a higher fidelity in style transfer.

**Table 4:** Comparison of network training duration.

Study	Duration(h)	Epochs
Ghiasi et al. [41]	20.45	2,000
Hybrid Garden StyleNet	15.30	2,000

The aesthetic style transfer of the virtual scenario in this study expands upon traditional 2D image style transfer techniques. It also broadens the medium of expression. This differs from previous style transfer research, which focused on videos and the textures and materials of 3D models. It also stands apart from studies on the surface shapes of 3D models [48, 49]. The analysis conducted in this study includes the following:

- Frame-by-Frame Stylization: Both existing video style transfer methods and the virtual scenario style transfer implemented in this study stylize each frame of the scenario before displaying it to the user, achieving a cohesive stylized expression for the entire scenario. However, a significant difference is that the virtual scenario style transfer in this study allows the input style image to be changed through user interaction while the application is running. This enables a dynamically changing aesthetic style for the scenario, enhancing the user experience of the virtual environment.
- High-Resolution Textures and Materials: Research on style transfer for textures and materials of 3D scenario models often achieves fine detail and high-resolution stylization, surpassing the resolution provided by the style transfer method in this study. These processes, however, are complex and computationally intensive, typically used for single 3D models or small, localized scenarios with few models. In contrast, the method used in this study enables fast style transfers for large scenarios or numerous models. The resolution of the stylized scenarios can reach 1080p, depending on the graphics card parameters, meeting practical application needs.
- Respecting Traditional Techniques: Traditional Chinese landscape painting artists often use techniques like exaggeration to depict subjects in a personalized way, influenced by their subjective intentions. These techniques do not fully represent the true shapes of objects. To prevent any misinterpretation of the appearance of cultural heritage, this study deliberately avoided focusing on the style transfer of 3D model shapes. Instead, the research concentrated solely on modifying the aesthetic style of the virtual scenario without altering the actual surface shapes of urban cultural heritage elements. The primary objective was to convey the aesthetic significance of the urban cultural heritage during its historical period, ensuring that users could appreciate its visual and cultural essence accurately.

The integration of style transfer algorithms enables the enrichment of virtual environments with historical and cultural context, bridging the gap between past and present. This technique not only preserves the visual integrity of cultural heritage sites but also revitalizes them by blending classical artistic elements with contemporary design innovations. The enhanced 3D models offer a dynamic and immersive experience, allowing users to interact with and appreciate the intricate details and historical significance of these sites.

Furthermore, the ability to dynamically alter the aesthetic style through user interaction ensures that the digital representations remain engaging and educational. This adaptability can be particularly useful for educational purposes, virtual tourism, and cultural exhibitions, where different styles can highlight various historical periods and artistic influences.

The application of style transfer algorithms in the digital preservation and restoration of urban cultural heritage contributes significantly to the field of cultural heritage management. It provides a versatile tool for conserving the aesthetic and historical essence of cultural sites while making them accessible to a broader audience through advanced technological means.

### Discussion

In this study, we propose and validated a hybrid model combining style migration with style prediction for the integration of traditional gardens with modern design elements. The experimental results show that the full model performs well in several key indicators, including style similarity, content retention, visual consistency and user satisfaction. These results demonstrate that the synergy between style migration and style prediction networks is crucial for generating high-quality, design-compliant images. Specifically, the full model reaches 0.92 and 0.94 in terms of style similarity and content retention, indicating that the model can effectively migrate the target style while maintaining the original image structure. Moreover, the visual consistency index (SSIM) and user satisfaction scores were higher than the other experimental configurations, indicating that the generated images had high quality and aesthetic feeling in both subjective and objective evaluations.

However, the experimental results also reveal some limitations of the model. For example, although removing style migration or style prediction components is able to reduce computational costs to some extent, it also results in a significant decrease in style similarity and visual consistency. This suggests that further optimization is still necessary in order to strike a balance between computational efficiency and generated image quality. In addition, although the model performs well in style migration effects, its stability in different combinations of styles and content images needs to be further improved to ensure consistent performance across a wider range of application scenarios.

From the perspective of application prospect, the hybrid model proposed in this paper has broad development potential, especially in the fields of cultural heritage protection, landscape design and aesthetic enhancement of virtual reality environment. By combining traditional art with modern design, the model can provide new technical means for the protection of digital cultural relics, and also provides a tool for designers and artists to explore new ways of aesthetic expression in the virtual environment. In the future, we will continue to optimize the computational efficiency and stability of the model in order to better cope with the complex requirements in practical applications and explore the potential applications of this technology in other fields.

## Conclusion

This study explores the potential of using style transfer algorithms to merge traditional garden aesthetics with modern design elements. By adopting a deep neural network model consisting of a style prediction network and a style transfer network, we successfully transferred the aesthetic features of traditional landscape paintings to a virtual scene of a classic garden. This approach provides a new idea for solving the problems of high cost, long cycle and poor repeatability in traditional garden design. Experiments in a virtual scene of the Humble Administrator’s Garden verify the effectiveness of our proposed method. The results show that the fusion of traditional and modern design elements through the style transfer algorithm can enhance the aesthetics and functionality of garden design, thereby contributing to the sustainability and cultural richness of the garden.

However, we are also aware that the virtual scenes and limited art styles used in the study may affect the breadth of the conclusions. Although this study demonstrates the feasibility and benefits of applying style transfer algorithms to virtual garden scenes, the applicability of these results still needs to be further verified under a wider range of garden types and diverse art styles. Therefore, in future studies, we will further explore the applicability of these methods in real scenes through the application of actual garden design projects and narrow the gap between traditional art and modern technology. We will also work with landscape architects, urban planners, and cultural heritage conservation experts to create garden spaces that are both visually appealing and sustainable and culturally meaningful.

In addition to garden design, this study also shows that style transfer algorithms can be extended to other fields of design and cultural heritage conservation, such as architecture, urban planning, and interior design. By using these algorithms, it is possible to create spaces that respect the historical context while incorporating modern technological advances.
